# Rationale for a 4-month, parallel-group, randomized controlled trial to assess the Feasibility and Efficacy of a Remotely delivered exercise training intervention for Hispanics/Latinos with Multiple Sclerosis (FERLA MS)

**DOI:** 10.1186/s40814-025-01641-5

**Published:** 2025-05-08

**Authors:** Victoria A. Flores, Stephanie L. Silveira, David X. Marquez, Dominique Kinnett-Hopkins, Augusto Miravalle, Fabian Sierra-Morales, Zulma Hernández-Peraza, Robert W. Motl

**Affiliations:** 1 Department of Kinesiology and Nutrition, University of Illinois Chicago, Chicago, IL USA; 2 Department of Management, Policy and Community Health, The University of Texas Health Science Center at Houston, Houston, TX USA; 3 School of Kinesiology, University of Michigan, Ann Arbor, MI USA; 4 Rush University Medical Center, Chicago, IL USA; 5 Department of Neurology, University of Illinois Chicago, Chicago, IL USA

**Keywords:** Hispanic, Multiple sclerosis, Health disparities, Feasibility, Health-related outcomes, Minority healthcare

## Abstract

**Background:**

Hispanic/Latino individuals with multiple sclerosis (MS) face greater disease burden and comorbidity due to limited healthcare access, underrepresentation in research, and social determinants of health (SDOH). Exercise training could manage health outcomes, but existing intervention research lacks Hispanic/Latino representation.

**Methods:**

We propose a feasibility and efficacy study of a theory-based, remotely delivered exercise training intervention for enhancing health outcomes in Hispanics/Latinos with MS. This study involves a randomized controlled trial (RCT) design and compares an MS-specific exercise training program with an active control condition over a 4-month period in a sample of 50 individuals who self-identified as Hispanic/Latino with MS. The sample will be recruited through MS networks and healthcare organizations serving a high proportion of Hispanics/Latinos with MS. The primary outcomes include feasibility metrics (i.e., process, resources, management, and scientific), secondary outcomes include potential effects of the exercise training program on health-related outcomes (i.e., physical and cognitive function, MS symptoms, and quality of life), and tertiary outcomes include the potential association of SDOH on feasibility and intervention efficacy on health-related outcomes.

**Discussion:**

The anticipated results of this study will provide evidence for the feasibility and initial efficacy of a remote exercise training intervention for Hispanics/Latinos with MS, a demographic often facing significant barriers to healthcare and rehabilitation. This research lays the groundwork for a fully powered RCT to support the efficacy of the approach and subsequent wider implementation. If successful, this project may significantly improve health and MS disease outcomes for Hispanics/Latinos with MS.

**Protocol version:**

April 7, 2025, Version 2; World Health Organization Trial Registration Data Set (see Appendix 1); SPIRIT Checklist (see Appendix 2).

**Trial registration:**

ClinicalTrials.gov (NCT05998616).

**Supplementary Information:**

The online version contains supplementary material available at 10.1186/s40814-025-01641-5.

## Background

Multiple sclerosis (MS) is an immune-mediated, neurodegenerative disease of the central nervous system, affecting approximately 1 million adults in the United States (US) and leading to a spectrum of consequences from cellular damage to diminished quality of life [1]. Of note, the Hispanic/Latino community in the US constitutes a significant segment of the MS population, with MS incidence rates markedly higher in the US than in their countries of origin [2, 3]. Compared to other racial and ethnic groups, Hispanics/Latinos with MS have a younger age of onset and more severe disease course compared to non-Hispanic whites with MS [4], and further exhibit higher disability scores and worse MS symptom scores relative to African Americans and non-Hispanic whites with MS [5–7]. More specifically, Hispanics/Latinos with MS have worse scores in domains of cognition, depression, and anxiety than African Americans with MS, and worse scores in domains of walking, dexterity, spasticity, and pain than non-Hispanic whites [5].

Social determinants of health (SDOH, i.e., economic stability, education, healthcare access, neighborhood and built environment, and social and community context) may potentially exacerbate these differences [8, 9]. Education and health literacy are associated with adverse health behavior and differences in health care utilization [10]. Previous analyses revealed that the biggest predictors of lower cognitive scores in order of importance were having MS or clinically isolated syndrome, lower educational attainment, and self-identifying as Hispanic/Latino or African American [11]. Additionally, inequitable access to healthcare for MS has led to longer delays in diagnosis for Hispanic/Latino patients compared to non-Hispanic white patients in the public healthcare system [12].

Hispanics/Latinos with MS are underrepresented in randomized controlled trials (RCTs) of disease modifying medications [13], and this lack of racial and ethnic diversity can influence risk patterns and treatment recommendations as well as limit the applicability and generalizability of findings. In 44 Phase III clinical trial publications, 38% did not report race or ethnicity, 31% reported race and ethnicity as a proportion of White participants only, and 31% reported two or more races/ethnicities [13]. More alarming is the significant lack of inclusion in RCTs focused on rehabilitation and exercise training [14–16]. One review paper identified that fewer than 10% of the 4280 persons with MS enrolled in RCTs of rehabilitation identified as Hispanics/Latinos [16]. We have reported an even worse omission in exercise training interventions for MS, where none of the 2034 enrolled persons across 53 studies with MS identified as Hispanic/Latino (15).

Omission may be due to previously observed beliefs that Hispanics/Latinos were a low MS risk population [17], poor trial design (i.e., no racial or ethnic sampling), or individual (i.e., economic stability, education, healthcare access) and structural (neighborhood/built environment, social/community context) SDOH. For example, previous analyses assessing barriers to MS research participation observed that Hispanics/Latinos with MS were not fully informed about the research trial, and Hispanics/Latinos with MS were more concerned about receiving poor-quality medical care, being taken advantage of by the research team, and risking losing their job or legal status than non-Hispanic whites [18]. Though reasons for lack of inclusion are multifactorial, this situation creates an evidence-based care void for Hispanics/Latinos with MS, particularly when considering exercise training as a form of rehabilitation and symptom management.

Exercise training is a promising second-line therapy associated with disease-modifying effects and improved physical and mental health outcomes for persons with MS [19]. We recently reported that Hispanics/Latinos with MS have significantly lower rates of physical activity than the general population [20]. Our analyses identified several MS outcomes (e.g., mobility, fatigue, cognition, and depression) that may be associated with lower physical activity rates [20]. Additionally, there is inequitable access to healthcare services for Hispanics/Latinos that may prevent access to physician-supervised programs for those living with MS [21]. These barriers to care include insurance coverage, immigration status, language and degree of health literacy, or mistrust of the healthcare system. Notably, Hispanics/Latinos with a neurological condition were 40% less likely to consult an outpatient neurologist [22], and exhibited a 49% higher level of medical mistrust compared to non-Hispanic whites [23].

We believe that the Guidelines for Exercise in Multiple Sclerosis (GEMS) approach [24], a home-based exercise training program with embedded behavioral approaches, may be promising for increasing physical activity in Hispanics/Latinos with MS. This program has been established as feasible, safe, and efficacious for increasing exercise behavior in inactive, non-Hispanic African Americans with MS [25]. GEMS is now undergoing a Phase III trial for African Americans with MS who have walking dysfunction and mobility disability [26]. These trials have set the stage for the delivery of a targeted version of GEMS for Hispanics/Latinos, as there are currently no existing exercise training interventions for this population with MS.

We proposed the Feasibility and Efficacy of Exercise Remotely delivered for Hispanics/Latinos with MS (FERLA MS) study as a salient opportunity for timely and successful recruitment of an underrepresented racial and ethnic minority population with MS into a RCT of exercise training. Our proposed pilot study focuses on a comprehensive approach and considers the potential influence of SDOH by delivering the intervention and materials remotely in the participant’s local community. We build this research on our previous feasibility trials evaluating a targeted, home-based exercise program for non-Hispanic African Americans with MS [25] and recent cross-sectional analyses of physical activity patterns and outcomes among Hispanics/Latinos with MS [20, 27]. This investigation is significant considering the current research landscape that emphasizes the need for systematic focus on race and ethnicity in MS trial designs [8, 16, 28], assessment of the impact of SDOH on MS [9, 29], and prescription of exercise as an effective treatment for MS symptoms [30]. This is particularly salient for researchers and clinicians, as a targeted exercise intervention can be delivered remotely through ongoing clinical care provided by MS healthcare providers. The provision of a carefully designed trial with a remotely delivered exercise program can efficiently address health disparities and improve physical and mental health outcomes in this marginalized community.

FERLA MS is a Phase-Ib study that evaluates a 4-month, remotely delivered exercise intervention for Hispanics/Latinos with MS using a single-blind, parallel-group, RCT design. The primary aim assesses the feasibility of this intervention, determined by measures of process (recruitment rate of ~ 70%, refusal rate of ~ 20%, retention rate of ~ 80% of 50 enrolled participants through the duration of the study, and an adherence rate of 75% to the intervention per participant), resource (total time and cost requirements), management (data collection, analysis, and storage), and scientific (detailed safety procedures and adverse events). The secondary aims assess the potential effects of the intervention on health-related outcomes (physical and cognitive function, symptoms of fatigue, anxiety and depression, exercise behavior, and quality of life), and the tertiary aim assesses the potential association of SDOH (economic stability, education, healthcare access, neighborhood and built environment, and social and community context) on the feasibility and efficacy of the remote intervention for improving health outcomes.

## Methods

### Study design and overview

The FERLA MS study is funded by the Chicago Chronic Condition Equity Network, a recipient of the National Institute of Minority Health and Disparities research grant for chronic disease prevention and treatment. The study will be undertaken by the Exercise Neuroscience Research Laboratory (ENRL) at the University of Illinois at Chicago (UIC) and conducted using a parallel group RCT design. The RCT examines the feasibility and efficacy of a remotely delivered exercise training program [26] compared with an attention/social contact, active control condition (stretching and flexibility) among Hispanics/Latinos with MS residing in the Chicagoland region and nearby communities of Illinois. The primary outcome is feasibility, including process, resource, management, and scientific measures. The secondary outcome is program efficacy on health-related outcomes, and the tertiary outcome is the potential effect of SDOH on feasibility and potential efficacy.

**Fig. 1:**
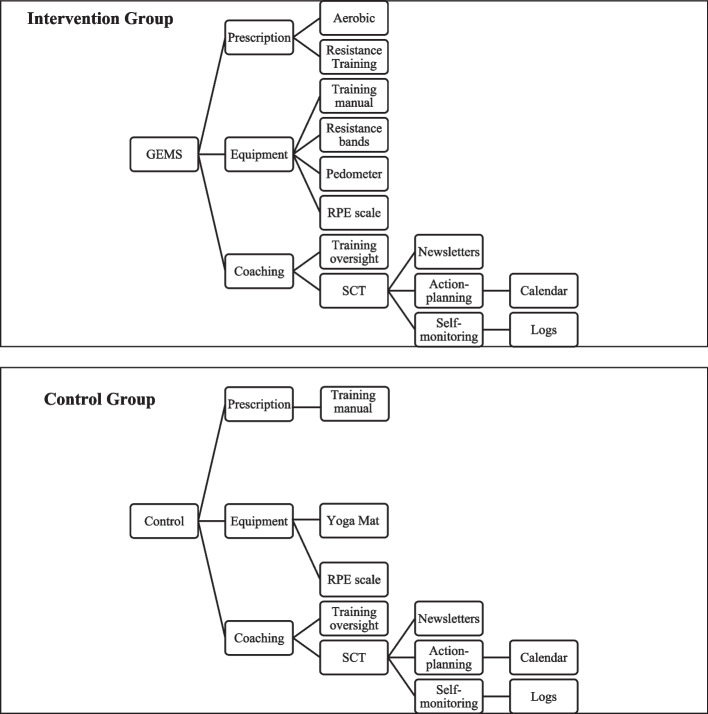
Intervention and control outline of the Guideline for Exercise in Multiple Sclerosis (GEMS) program. *Note.* GEMS = Guidelines for Exercise in Multiple Sclerosis; RPE = rate of perceived exertion; SCT = social cognitive theory

Participants (*N* = 50) will be randomly assigned by a research member uninvolved in intervention delivery and outcome assessment into conditions using a random numbers sequence with concealed allocation. The conditions will be administered for 4 months by a trained behavioral coach, uninvolved in randomization and outcome assessment. All outcomes will be assessed at baseline and immediately after the 4-month intervention by a treatment-blinded outcomes assessor. The outcomes of interest will be monitored before and after the 4-month intervention period and data analysis will involve intent-to-treat principles and linear mixed models. The study does not include a data monitoring committee as it is a low risk, behavioral intervention in a population that is not identified as vulnerable and has been approved by the UIC Institutional Review Board (UIC IRB) (2023–0665). The study will consist of an exercise intervention group that completes aerobic and resistance training exercises, and an active control group that completes flexibility training exercises. Both will follow the GEMS protocol [24] with materials consisting of a program manual, exercise prescription, equipment, social-cognitive theory (SCT) based educational materials such as newsletters, logbooks, and calendars, and one-on-one behavioral coaching provided by the trained behavioral coach (Fig. 1).

### Participant recruitment, sample size, and screening

The target sample is 50 persons (25 per intervention and control group) who self-identify as Hispanic/Latino with MS living in the Chicago and nearby neighborhoods of Illinois in the US. The sample size was selected based on considerations of feasibility, precision, and funding. For a Phase-Ib feasibility RCT, a small sample size is appropriate to explore process outcomes, intervention delivery, and adherence [31]. The chosen target of 50 participants will allow for adequate evaluation of recruitment flow, retention and adherence rates, and other feasibility metrics while accounting for potential attrition. This is consistent with pilot study recommendations suggesting that approximately 10–20% of the expected sample size for a confirmatory trial is sufficient to estimate feasibility parameters with reasonable precision [32]. For example, in a future RCT requiring 100 participants (assuming an effect size of 0.3, 80% power, and a two-sided alpha of 0.05), a pilot sample size of 10–20 participants would meet these recommendations. The target sample of 50 participants for FERLA MS exceeds this range, representing approximately three times the minimum recommended sample size for a feasibility study (16 participants). This larger sample size ensures sufficient data to assess recruitment and adherence rates, monitor AEs, and refine the intervention design for planning a future RCT.

Participant recruitment began on March 18th, 2024, and the first wave started on April 22nd, 2024. Subsequent waves will continue at 1 to 2-month intervals with recruitment ending on February 1st, 2025. Recruitment will occur primarily through the distribution of advertisements through the National Multiple Sclerosis Society (NMSS), Consortium of Multiple Sclerosis Centers, the North American Registry for Multiple Sclerosis, and iConquerMS and supplemented as necessary. Advertisements will describe the study as comparing two exercise approaches for managing symptoms of MS and improving health-related outcomes for Hispanics/Latinos with MS. Interested individuals will contact the project coordinator via email or a toll-free telephone number. The project coordinator will then conduct a follow-up phone call to explain the study and its procedures, answer any questions, screen for inclusion and exclusion criteria, and, if eligible, gather the necessary information to obtain informed consent.

### Eligibility criteria

Interested individuals will be assessed for the following inclusion criteria: (1) age between 18 and 65 years, (2) self-reported diagnosis of MS, (3) relapse-free for at least 30 days, (4) able to walk with or without an assistive device, (5) insufficient physical activity (i.e., not meeting current physical activity guidelines of 150 min of moderate to vigorous physical activity per week), (6) willingness to complete all required testing procedures, outcome questionnaires, and randomization, (7) self-identify as Hispanic/Latino, (8) able to speak, read, and understand English, (9) currently reside in Chicago, (10) access to the internet and email, and (11) safe for exercise based on Physical Activity Readiness Questionnaire [33].

### Primary outcomes

The primary outcome is the feasibility of the remotely delivered exercise training intervention. The pilot will follow the proposed metrics from GEMS [24]. The four key metrics (i.e., process, resources, management, and scientific) were based on major categories of feasibility that align with methods for collecting and assessing relevant data, and the importance of each feasibility metric to the development of future trials [24]. All metrics, their assessments, potential thresholds, and importance are displayed in Table 1.

**Table 1:** Feasibility metrics, assessments, thresholds, and importance

Metric	Monitored and assessed	How this will be monitored and assessed	Thresholds/importance
Process: assesses participant recruitment and retention	a. Recruitment and refusal rates b. Retention and adherence rates	a. We will use USPS, phone and electronic mail, and the pre-enrollment form online to record all contact with potential participants’ acceptance or refusal reasons to join the intervention. We will record all participants’ flow through the recruitment, enrollment, intervention, and post-intervention sections of the study b. We will record adherence with the intervention via logbooks and video chats, and during follow-up assessments	a. Recruitment rate of ~ 70% and refusal rates of ~ 20%. This will provide information on optimal recruitment methods and reason for refusal b. Retention rate of ~ 80% and adherence rates of ~ 75% per participant. This will provide target areas for optimizing participant retention and intervention adherence
Resource: assesses communication and monetary requirements of the study	c. Communication time with participants d. Communication needs of participants and staff e. Monetary costs of research	c. We will utilize a password protected database to monitor contact time with all potential and enrolled participants d. We will record all communication problems and alterations with participants and staff e. We will record all monetary costs for the study; for both the intervention and control groups	c. None; this will provide information on communication frequency, quality, and highlight communication problems d. None; this will establish communication needs and anticipated communication problems e. None; this will establish monetary costs to conduct the research and establish areas for cost saving
Management: assesses data management including collection, analysis and storage	f. IRB protocol procedures and amendments g. Participant data collection, analysis, and storage	f. We will document and record time required for IRB protocol development and submission, and any follow-up amendments and modifications to the approved procedures g. We will document the time needed to record, clean, analyze, and store data in UIC Qualtrics, Redcap, Microsoft Excel, and SPSS Statistics	f. None; this will establish staff preparation time for IRB protocol development and amendments g. None; this will establish anticipated time needed for data management prior to analysis
Scientific: assesses trial safety, handling of AEs, SAEs, and clinical emergencies, and participant burden	h. Reporting and handling AEs, SAEs and clinical emergencies i. Number of AEs, SAEs, and clinical emergences j. Participants’ experienced burden throughout the program	h. We will follow standard University IRB protocol to report all AEs, SAEs, and clinical emergencies, and ask participants to report and record all medical concerns via logbooks and video chats i. We will record the number of AEs, SAEs, and clinical emergencies experienced by and reported from participants in both intervention and control groups j. We will record participant feedback on the study during video chats, and in a post-intervention interview. We will also use participant self-reporting of total exercise time and rate of perceived exertion during each exercise session, reported via logbooks and video chats	h. None; this will detail what safety procedures should be implemented and establish any medical concerns participants would have i. None; this will determine the safety and feasibility of the intervention and highlight safety considerations j. None; this will determine acceptability and highlight considerations for alterations. This will also determine compliance and further allow correct conclusions to be drawn from the results

*USPS* United States Postal Service, *IRB* Institutional Review Board, *UIC* University of Illinois Chicago, *SPSS* Statistical Package for the Social Sciences, *AE* adverse event, *SAE* serious adverse event

Process measures will be evaluated through recruitment and refusal rates, tracked via interactions with potential participants by phone, email, and our online pre-enrollment form. These interactions will be recorded to monitor recruitment flow using recorded interactions, participant logbooks, and attendance at check-ins throughout the 4-month program, with thresholds set at ~ 70% for the recruitment rate, ~ 20% for the refusal rate, ~ 80% for the retention rate, and ~ 75% for the adherence rate per participant. Process measures will inform optimal recruitment methods and reasons for refusal, as well as target areas for improving participant retention and adherence to the intervention.

Resource measures will be evaluated by frequent communication with participants and staff, noting barriers, needs, and monetary requirements of the study. We will utilize a password protected database to monitor contact with all potential and enrolled participants, record all problems with communication alterations, and record all monetary costs for the study for both the intervention and control groups. Resource measures will provide information on best communication frequency practice such as preference of communication through either phone or UIC Zoom, as well as highlight anticipated communication problems for future trials and cost requirements. Management will assess time required for University IRB procedures and data handling. We will document the time required for IRB protocol development and submission, and follow-up amendments and modifications. We will record the time needed to record, clean, analyze, and store data using UIC Qualtrics and Redcap, Microsoft excel, and SPSS in UIC’s encrypted server. This will establish staff preparation and report time for IRB procedures and accuracy in data collection/entry.

Details of data confidentiality, management, and storage are outlined in IRB# 2023–0665 procedures. Upon enrollment, participants will be assigned a coded participant identification number, and all collected data will be stored on UIC’s encrypted server and UIC Qualtrics and RedCap. We will document all communications between the IRB and staff, time from submission of IRB application to approval and necessary modifications. We will document call date and time, attempted call time, communication mode (via Zoom or phone), and duration of chat for each participant during the intervention. We will check data completeness and record our handling of standard university protocol for reporting of adverse events (AEs), serious adverse events (SAEs), and clinical emergencies. The research team will meet monthly to assess trial conduct and data management.

Scientific measures will evaluate trial safety and participant burden. We will document all AEs and SAEs, and clinical emergencies, adhering to standard UIC IRB protocols. Unblinding of participants will only be permissible for reporting AEs, SAEs, and clinical emergencies. Participants will be instructed to discuss any medical issues during video chats with the behavioral coach. Feedback on their experiences and adherence will also be collected after the post-intervention assessment. This will inform intervention safety and practicality, guide future modifications, and provide data for power calculations and potential clinical impact.

### Secondary outcomes

The secondary end-points are remotely assessed physical and cognitive function, symptoms, exercise behavior, and quality of life. Physical function will be remotely assessed with the 30 s sit-to-stand (30STS) [34], and cognitive function will be remotely assessed with the Symbol Digit Modalities Test (SDMT) [35] and the California Verbal Learning Test II (CVLT-II) [36]. Symptoms, including fatigue, anxiety, and depression will be assessed with the Fatigue Severity Scale (FSS) [37] and the Hospital Anxiety and Depression Scale (HADS) [38]. Exercise behavior will be assessed with the Godin Leisure-Time Exercise Questionnaire (GLTEQ) [39], and health-related quality of life will be assessed with the Short-Form 12-Item Health Survey (SF-12) [40].

In the 30STS, participants will be instructed to complete as many stands as possible within 30 s, with arms crossed and held against the chest, fully sitting between each stand. The score will be the total number of stands performed [34]. The SDMT assesses visual processing speed, and will require participants to verbally identify digit-symbol pairings as quickly as possible in response to a series of unpaired symbols displayed on screen. The outcome is the total number of correct responses in 90 s, and it is recognized as a sensitive test for slowed information processing in MS [36]. The CVLT-II will assess verbal learning and memory, in which participants will be read aloud 16 words and will immediately recall as many words as possible, in any order, for each of the five trials. The total score out of 80 will be calculated by summing the number of correct responses from each trial [35]. The FSS is a 9-item questionnaire that measures subjective fatigue and its impact on individuals [37]. A score above 4 indicates severe MS-related fatigue, and a change of 1.9 represents the minimal detectable change at 95% confidence interval [41]. The HADS is a 14-item questionnaire that measures symptoms of depression and anxiety separately. Scores for anxiety and depression subscales range from 0 to 21 and are classified as “normal” (0–7), “mild” (8–10), “moderate” (11–14), and “severe” (15–21). The total score ranges from 0 to 42, with higher scores indicating greater anxiety and depressive symptoms [38].

The GLTEQ will assess exercise behavior. Participants will record the number of bouts of mild, moderate, and strenuous physical activity lasting more than 15 min in a typical week. Overall leisure activity score will be calculated by multiplying the recorded number of 15-min bouts of physical activity by weights of 3, 5, and 9, respectively, and then adding the values to obtain a score between 0 and 119, with higher scores indicating higher levels of physical activity [39]. The SF-12 is a shortened version of the short-form 36-item health survey, which measures overall health status. The questionnaire consists of 12 items that assess eight different dimensions (i.e., physical functioning, role-physical, role-emotional, mental health, bodily pain, general health, vitality, and social functioning), and provides both a physical and mental component summary score. The component scores are adjusted based on a relative average, where a score above 50 is considered above average and a score below 50 is considered below average [40].

### Tertiary outcomes

The tertiary end-points are the potential association of SDOH factors on feasibility and efficacy of the intervention, and assessed with the PhenX Toolkit, a consensus measurement for phenotypes and exposures in human research [42]. The instrument will assess individual and structural determinants of health through 20 aspects. Individual SDOH will include birthplace, biological sex, gender identity, sexual orientation, race and ethnicity, education, income, current employment, and racial/ethnic discrimination in recent lifetime. Structural SDOH will include health insurance coverage, health services, disparate health care, discrimination in health care, food insecurity, health literacy, sanitation access, job insecurity, spirituality, religious behaviors, and physical activity in neighborhood environment.

### Manipulation check

To ensure the efficiency of the intervention and verify participant engagement, we will implement manipulation checks to confirm that the intervention is being perceived and executed correctly. Participant engagement will be monitored through ongoing video chats and telephone calls with the behavioral coach where progress, challenges, and adherence to the exercise regimen will be discussed and documented. Participants will be encouraged to maintain detailed exercise logs, noting the duration, type, and intensity of each session. Cognitive and physical assessments will be conducted at baseline and post-intervention to determine the intervention’s impact on physical and cognitive functions. Additionally, exit interview questions at the end of the program will provide qualitative feedback on participants’ experiences, understanding, and perceptions of the intervention, as well as suggestions for improvement. No explicit post-intervention care will be required for this low-risk behavioral intervention.

### AEs and program adherence

We will closely monitor AEs and SAEs through regular virtual check-ins with participants throughout the study. Participants will also be encouraged to reach out to the project coordinator or behavioral coach for any AE that occurred outside check-ins. Adherence to the program will be tracked by recording the number of sessions completed, while compliance will be assessed by evaluating the number of sessions completed as prescribed. Both adherence and compliance data will be collected via participant log entries over the 4-month intervention period.

### Procedures

The study procedures are modeled after previous research [24, 26], and has been reviewed and approved by the UIC IRB (2023–0665) and registered at ClinicalTrials.gov (NCT05998616) [26]. Interested participants will contact the project coordinator, who will describe the study using an IRB-approved script and conduct a phone screening for inclusion criteria. Participants will review and sign the consent form electronically with the project coordinator, and once enrolled, be scheduled for baseline data collection with outcome assessors uninvolved in delivering the intervention. The project coordinator will confirm participants are comfortable using the UIC Zoom link provided with their device of choice (tablet, desktop, laptop, or phone) for physical and cognitive assessments. Participants will also be provided with a link to complete questionnaires via UIC Qualtrics and UIC RedCap. The questionnaires will take approximately 30–60 min to complete, and participants will have the opportunity to save and return where they left off.

Once participants complete baseline assessment and questionnaires, they will be randomly assigned to either the intervention (exercise training) or control (flexibility) group using a random number sequence with concealed allocation (opaque sealed envelopes) by a research team member uninvolved in outcome assessment and intervention delivery. Both conditions will be delivered in 5–10 waves of 5–10 participants each, over a 4-month period by one behavioral coach to reduce variance in intervention delivery and who will be uninvolved in outcome assessment. Participants will be instructed to contact the project coordinator or behavioral coach via a toll-free phone or study email in case of an AE or any other issue. Participants will complete the same physical and cognitive assessments and questionnaires immediately following the 4-month intervention. Additionally, formative feedback will be collected through an exit interview with outcome assessors uninvolved in randomization and intervention delivery, and both intervention and control groups will be asked the same open-ended questions to identify opportunities for improving the intervention.

### Intervention condition

The intervention incorporates the GEMS program for adults with MS aged 18 and older and who can walk with and without an assistive device (Table 2). The intervention will be delivered remotely through teleconferencing, either via phone or UIC Zoom, and will be completed within the participant’s home and/or community. The aerobic exercise component will focus on moderate-intensity walking, with progression individualized based on three different trajectories established from the GEMS protocol [24]. Participants will begin with 10 min of walking per day for the first 2 weeks, followed by an additional 14 weeks with progression trajectories tailored to achieve the minimal dose within 6, 8, or 10 weeks. The intensity will be set at a step rate of 100 steps per minute, and exercises can be performed at home and/or community. Resistance training will be conducted using elastic resistance bands. The training manual will provide detailed instructions and video demonstrations for each exercise, along with additional challenges and modifications to ensure safety and progression. The program will offer three different trajectories for individualization, ensuring that each participant can safely progress according to their capabilities.

**Table 2:** Description of the guidelines for exercise in multiple sclerosis program features

Feature	Intervention description
Prescription	Prescriptive guidelines for exercise in multiple sclerosis (MS) Individually tailored based on level of comfort/trainability
Frequency	3 times per week
Exercise session duration	Approximately 1 h
Exercise session intensity	Moderate 100 steps/min (pedometer) Modified Rate of Perceived Exertion (RPE) between 4 and 6
Intervention length	16 weeks
Meeting with coach ◦ Weeks 1–4 ◦ Weeks 5–8 ◦ Weeks 9–16	Weekly Bi-weekly Monthly
Setting	Home and/or community
Supervision ◦ Who ◦ Mode	Trained personnel Remote, teleconferencing
Exercise modes	Aerobic—Walking Resistance—Bands and body weight
Materials provided	Program manual Newsletters Logbook Calendar Online exercise video demonstrations
Equipment provided	Pedometer Bands
Training oversight	Zoom/Telephone
Behavioral intervention	Zoom/Telephone
Intervention safety	NMSS resources Fall prevention instructions Zoom/Telephone oversight and AE/SAE reporting

*NMSS* National Multiple Sclerosis Society, *AE* adverse event, *SAE* serious adverse event

### Exercise prescription

The GEMS exercise prescription follows current guidelines for adults with MS aged 18 and older who have mild-to-moderate disability (Table 3) [43]. The guidelines provide a framework for safely and efficiently engaging in exercise training to achieve fitness and health benefits. The guidelines recommend 30 + min of moderate-intensity aerobic exercise two to three times per week and resistance training targeting major muscle groups two to three times per week. Based on a meta-analysis indicating that this frequency has the greatest impact on MS symptoms [44], we opted for 3 days per week of both aerobic and resistance exercise. These sessions can occur on the same day, but participants should have 1 day of rest between exercise sessions to ensure proper recovery and a balanced exercise regimen.

**Table 3:** Recommended exercise prescription for adults with mild to moderate MS-related disability

Exercise training parameter	Aerobic prescription	Resistance prescription
Frequency—How often?	3 days per week	3 days per week
Duration—How much?	Gradual increase in the duration of exercise from 10 to 30 min over time	The exercise bouts should range from 1 to 3 sets between 10 and 15 repetitions per exercise
Intensity—How hard?	These activities should be performed at a moderate intensity—between 4 and 8 on the 10-point RPE scale	Pick a resistance that you can finish 10–15 repetitions of the last set comfortably
Mode—How too?	Walking (over-ground or treadmill)	Resistance training activity mainly target major/large muscle groups (elastic bands)
Special considerations	Overall progression should start with either duration or frequency, and finally progress intensity per tolerability of the person Rest your muscles for 1–2 min duration in between sets and muscle groups Rest your muscles for at least 1 day between strength training sessions Aerobic and resistance training can be performed on the same day as aerobic exercise training, depending on tolerability MS-specific symptoms (i.e., fatigue and heat sensitivity) should be identified and discussed before prescribing an exercise routine	

*RPE* rate of perceived exertion

### Aerobic exercise

The aerobic exercise training will focus on 30 + min of moderate-intensity walking, performed 3 days per week. Walking is the most common exercise for people with mild MS that can be administered and monitored in non-supervised environments. The progression of the aerobic exercise duration will follow three different trajectories for individualization (Table 4). All participants will start with the same duration of aerobic exercise for the first 2 weeks, consisting of 10 min of walking per day, 3 days per week. This initial 2-week period serves as an adjustment phase and a reference point for determining the appropriate trajectory for the remaining 14 weeks of the program. There will be three progression trajectories (orange, blue, and green) where the minimal dose of aerobic exercise will be achieved in 6, 8, or 10 weeks, respectively. The intensity of the aerobic exercise will be based on a step rate of 100 steps per minute, which corresponds to moderate-intensity exercise for persons with MS and other conditions [45]. Participants can complete the aerobic portion of the intervention at home or in the community (e.g., park, shopping mall, high school track).

**Table 4:** Progression of the 3 levels or trajectories of progression for the GEMS program

Week	Orange		Blue		Green	
	Aerobic training	Resistance training	Aerobic training	Resistance training	Aerobic training	Resistance training
	Phase I					
**1**	10 min, ~ 1000 steps			1S, 10R, 5E		
**2**	10 min, ~ 1000 steps			1S, 10R, 5E		
	Phase II					
**3**	15 min, ~ 1500 steps	1S, 15R, 5E	15 min, ~ 1500 steps	1S, 12R, 5E	10 min, ~ 1000 steps	1S, 12R, 5E
**4**	20 min, ~ 2000 steps	2S, 10R, 5E	15 min, ~ 1500 steps	1S, 15R, 5E	15 min, ~ 1500 steps	1S, 12R, 5E
**5**	25 min, ~ 2500 steps	2S, 12R, 5E	20 min, ~ 2000 steps	2S, 10R, 5E	15 min, ~ 1500 steps	1S, 15R, 5E
**6**	30 min, ~ 3000 steps	2S, 15R, 5E	20 min, ~ 2000 steps	2S, 10R, 5E	20 min, ~ 2000 steps	2S, 10R, 5E
**7**	30 min, ~ 3000 steps	2S, 15R, 6E	25 min, ~ 2500 steps	2S, 12R, 5E	20 min, ~ 2000 steps	2S, 10R, 5E
**8**	30 min, ~ 3000 steps	2S, 15R, 6E	30 min, ~ 3000 steps	2S, 15R, 5E	25 min, ~ 2500 steps	2S, 12R, 5E
**9**	30 min, ~ 3000 steps	2S, 15R, 7E	30 min, ~ 3000 steps	2S, 15R, 6E	25 min, ~ 2500 steps	2S, 12R, 5E
**10**	30 min, ~ 3000 steps	2S, 15R, 7E	30 min, ~ 3000 steps	2S, 15R, 6E	30 min, ~ 3000 steps	2S, 15R, 5E
**11**	30 min, ~ 3000 steps	2S, 15R, 8E	30 min, ~ 3000 steps	2S, 15R, 7E	30 min, ~ 3000 steps	2S, 15R, 6E
**12**	30 min, ~ 3000 steps	2S, 15R, 8E	30 min, ~ 3000 steps	2S, 15R, 8E	30 min, ~ 3000 steps	2S, 15R, 6E
**13**	30 min, ~ 3000 steps	2S, 15R, 9E	30 min, ~ 3000 steps	2S, 15R, 8E	30 min, ~ 3000 steps	2S, 15R, 7E
**14**	30 min, ~ 3000 steps	2S, 15R, 9E	30 min, ~ 3000 steps	2S, 15R, 9E	30 min, ~ 3000 steps	2S, 15R, 8E
**15**	30 min, ~ 3000 steps	2S, 15R, 10E	30 min, ~ 3000 steps	2S, 15R, 10E	30 min, ~ 3000 steps	2S, 15R, 9E
**16**	30 min, ~ 3000 steps	2S, 15R, 10E	30 min, ~ 3000 steps	2S, 15R, 10E	30 min, ~ 3000 steps	2S, 15R, 10E

*S* number of sets, *R* number of repetitions, *E* number of exercises

### Resistance exercise

The strength training will consist of 1–2 sets of 10–15 repetitions of 5–10 exercises targeting the lower body, upper body, and core muscle groups, performed 3 days per week. The lower body exercises include chair squats, calf raises, glute bridges, forward lunges, leg extensions, hamstring curls, hip abductions, and glute kickbacks. The upper body exercises include back rows, bicep curls, shoulder front raises, tricep extensions, overhead presses, chest presses, lateral raises, and reverse flies. Core exercises include abdominal curls, standing ab twists, and modified planks. Modifications will be suggested to ensure safety, such as performing shoulder front raises while seated or holding onto a sturdy chair during calf raises or forward lunges. The progression of resistance exercise training (sets, repetitions, and exercises) will follow three different trajectories for individualization (Table 4). All participants will start with the same number of sets and repetitions of 5 exercises for the first 2-week period, consisting of one set of 10 repetitions of chair squats, shoulder front raises, bicep curls, abdominal curls, and calf raises per session. This initial 2-week period serves as an accommodation phase and a benchmark for selecting a trajectory for the remaining 14 weeks of the program. There will be three progression trajectories (orange, blue, and green) where the minimal dose of resistance exercise will be achieved in 6, 8, or 10 weeks, respectively. The intensity will be controlled with a standardized resistance band for the first 2 weeks, after which participants can use a self-selected resistance band that allows 10–15 repetitions with proper technique for the remaining 14 weeks. All participants will undertake the resistance program with the provided elastic bands at home.

### Exercise equipment—pedometer

Participants will be provided with a CW-300 Yamax Digi-Walker™ pedometer (New-Lifestyles, Inc., Lees Summit, MO, USA) worn on the waistband to monitor and track walking intensity based on steps per minute. The CW-300 was selected for its simplicity and accuracy in measuring steps, particularly during slow walking. Participants will monitor exercise intensity using a step rate of 100 steps per minute, as measured by the pedometer, based on evidence that 100 steps per minute is the threshold for moderate-to-vigorous intensity exercise in MS. Initially, participants will undertake a 20-step test to confirm the proper placement of the pedometer for accuracy. They will then wear the pedometer in the correct location to monitor and track aerobic exercise intensity. During the first six sessions over the initial 2 weeks, participants will check the pedometer every 5 min of walking to ensure it reads approximately 500 steps after the first 5 min and 1000 steps after 10 min. If the reading is more than 50 steps off in either direction per 5-min increment, participants should adjust their speed accordingly. After this initial accommodation period, participants will increase the step increment by 500 steps for every additional 5 min of aerobic exercise per session.

### Exercise equipment—resistance bands

Participants will receive elastic resistance bands from Black Mountain Products (McHenry, IL, USA) for their resistance training exercises. These bands were chosen for their versatility, portability, affordability, and minimal storage requirements. Using resistance bands can provide similar benefits to traditional resistance training equipment like free weights and machines, which aligns with MS exercise guidelines. The set includes five bands with varying resistance levels (2–30 lbs), a door anchor, an ankle strap, and a carrying case. Participants will start with the lightest resistance band (2–4 lbs). After the initial 2-week training period, participants will work with the behavioral coach to choose their resistance level, ensuring it allows for 10–15 repetitions with proper technique. They will be instructed to complete the repetitions consecutively, with a 1–2-min rest between sets. Each participant will receive a training manual with detailed instructions and demonstrations of the exercises and modifications and a QR code for video demonstrations of the exercises. The behavioral coach will offer additional guidance and/or modifications remotely via UIC Zoom teleconference.

### One-on-one coaching

The GEMS program includes one-on-one semi-structured coaching sessions with a behavioral coach. These sessions focus on providing exercise training guidance and oversight, discussing behavioral strategies such as action planning and self-monitoring, and presenting and discussing newsletters based on SCT to optimize exercise compliance. The primary objective of these coaching sessions is to offer guidance and oversight into exercise training. Behavioral coaches receive standardized training from experts in exercise prescription and behavior change for physical activity before working with research participants. The sessions are designed to provide feedback and information on appropriate exercise techniques and strategies, as well as progression through the exercise program. Sessions are scheduled in weeks 1–3, and weeks 5, 7, 11, and 15 of the 4-month program (see Appendix 2). Participants can choose from three levels of exercise difficulty, which they will discuss with the behavioral coach based on their experiences during the first 2 weeks of the program. These difficulty levels vary in terms of progression toward meeting exercise guidelines, accommodating the various levels of ability in persons with MS. The coaching sessions also emphasize action planning and self-monitoring, along with the delivery and discussion of SCT constructs through content-relevant newsletters. The one-on-one sessions will be conducted via a Health Insurance Portability and Accountability Act (HIPAA)-compliant teleconferencing system (UIC Zoom). If a participant does not have internet access, video chats will be replaced with telephone calls.

### Action planning and self-monitoring

Action planning and self-monitoring are crucial for the long-term success of the GEMS program, as they help maximize behavior change and compliance with the intervention. Participants will be provided with calendars to schedule their aerobic and resistance training sessions. These calendars can also be used to plan exercise sessions around other life events, such as doctor visits or vacations, and serve as a memory aid for future events. Self-monitoring will be facilitated through exercise adherence logs, which participants will use to track their exercise sessions over the 4-month intervention. These logs will allow participants to record missed sessions and the reasons for them, document the completion status of resistance training exercises, and note step information from the pedometer after each exercise session.

### Newsletters

Participants will receive newsletters on a pre-determined schedule throughout the program (weeks 1–3, 5, 7, 11, and 15; see Appendix 2. These newsletters will cover six SCT-based topics: Outcome Expectations, Self-Monitoring, Goal-Setting, Self-Efficacy, Barriers, and Facilitators (Table 5). Each newsletter will provide instructional material on the topic, links to websites for more information, reflections from previous participants, practical tips for exercise, and exercise-related things to try.

**Table 5:** Behavioral change session content

Week 1 Introduction to program	*Tele/video-chat 1:* clarification of materials received and initial questions; explanation of program; planning exercise schedule; using the logbook and qualtrics; *Newsletter 1:* exercise expectations; exercise outcomes
Week 2 Outcome expectations	*Tele/video-chat 2:* compliance with program; using the manual, logbook, and Qualtrics; Identifying personal outcomes
Week 3 Choosing a program	*Tele/video-chat 3:* compliance with program; comparison of orange, blue, and green programs; choosing a program; *Newsletter 2:* self-monitoring defined; benefits of self-monitoring
Week 4 Self-monitoring	*Tele/video-chat 4:* compliance with program; using your pedometer; understanding exercise intensity
Week 5 Goal-setting	*Tele/video-chat 5:* compliance with program; setting specific, measurable, adjustable, action-oriented, realistic, and time-limited (SMAART) goals; Performing resistance training exercises correctly; Tracking progress; *Newsletter 3:* exercise related SMAART goals defined
Week 7 Self-efficacy	*Tele/video-chat 6:* finding your self-confidence; what to do when you feel like quitting; involving family; *Newsletter 4:* self-efficacy defined; experiencing success, choosing role models, accepting encouragement and managing physical and emotional responses; reminder that program is specific for persons with MS
Week 11 Overcoming Barriers	*Tele/video-chat 7:* identifying your barriers; making plans to overcome obstacles; dealing with MS symptoms; *Newsletter 5:* exercise barriers defined; common barriers (facilities, social, and symptoms); strategies to overcome barriers
Week 15 Identifying facilitators	*Tele/video-chat 8:* how to keep going on your own; making adjustments as needed; setting future goals; *Newsletter 6:* exercise facilitators defined; common facilitators (having a goal, enjoyment, social support, knowledge); using facilitators long term

### Program pragmatics

After consent and randomization, the project coordinator will schedule participants’ baseline assessments with an outcome assessor. The coordinator will then mail a package containing the intervention materials (yoga mat and/or resistance bands and pedometer, a logbook, calendar, and an intervention-specific training manual) via United States Postal Service and schedule participants’ first one-on-one session with the behavioral coach. The first one-on-one session will serve as an orientation, during which the behavioral coach will confirm receipt of the study materials, gather more information about the participant’s motivations and experiences with MS and exercise, and provide an overview of the exercise program. Participants will be instructed to contact the behavioral coach via phone or email in the event of an AE or any other problem in addition to the scheduled chat sessions. After the 4-month intervention, the project coordinator will schedule participants’ post-intervention assessment and interview with an outcome assessor. The overall summary of the GEMS program is provided in Table 6. This is a comprehensive program with an exercise prescription appropriate for MS and individualized per participant, and with materials for home-based delivery and behavioral change intervention and support with implementation and compliance.

**Table 6:** Summary of the 4-month home-based aerobic and resistance exercise training prescription, materials, and behavior change interactions

Exercise prescription	
Home-based resistance and aerobic training	Based on the exercise training guidelines for adults. Aerobic-training; 10–30 min of moderate-intensity walking (1000 steps/10 min) performed 3 days a week. Resistance training; 1–2 sets, 10–15 repetitions of 1–10 exercises targeting lower body, upper body, and core muscle groups performed 3 days per week
Program variation	Orange—Aerobic and resistance training time and intensity increased over 16 weeks, and exercise guidelines achieved and maintained on week 6. Blue—as above, exercise guidelines achieved and maintained on week 8. Green—as above exercise, guidelines achieved and maintained on week 10
Materials	
Exercise equipment	Aerobic equipment; Yamax CW-300 Digi Walker™ pedometer. Resistance equipment; elastic resistance bands varying in 5 increments of strength
Online Videos	Demonstrations of resistance training exercises including modifications for differing levels of physical capability
Program manual	An introduction to the staff, safety information, guidelines for exercise in MS, description of equipment, and a detailed description of the resistance and aerobic exercises to be performed and how to progress over 4 months. Comparison tables of orange, blue, and green programs
Calendars	Four undated calendars to facilitate exercise planning
Logbook	Annotated booklet for participants to note exercise participation, feelings, and rating of perceived exertion (RPE) after each session. Aerobic training recorded per session as; length of time (in minutes) walked and number of steps per session, 5-point scale (stars) to describe how participants would rate the session, 100-point slider to describe enjoyment of exercise session, and RPE on 1–10 scale. Resistance training recorded per session as; number of sets, repetitions and resistance band utilized for the prescribed exercise per session, 5-point scale (stars) to describe how participants would rate the session, 100-point slider to describe enjoyment of exercise session, and RPE on 1–10 scale
Behavioral change interaction	
Tele-chats	Designed to provide participants with feedback and information on progression through the exercise program as well as to provide social accountability
Newsletter	Newsletters provide material on specific behavioral change content, information from other resources, testimonials of individuals who have experienced benefits of exercise, and tips for participants to try at home

### Intervention safety protocols

To ensure participant safety during the home-based exercise intervention, several precautions will be implemented, given the absence of direct supervision. Each participant will receive a detailed safety instruction sheet within their exercise manual. These guidelines include recommendations such as informing their healthcare provider of their participation in the FERLA MS program, warming up before exercise and cooling down afterward with a 2–5-min slow-paced walk or stretch, and choosing a safe exercise environment—free from slippery floors, poor lighting, crowded spaces, or other potential tripping hazards. Participants will also be advised to exercise near a stable surface, such as a wall, door frame, or large piece of furniture, to support balance if needed. Participants will be instructed to report any falls occurring outside of exercise sessions, unusual pain experienced during exercise, and updates regarding medications that might impact attention, balance, or concentration to the behavioral coach. Additionally, they will be encouraged to document any concerns or AEs in their exercise logbook. Logbook entries will be submitted through Qualtrics and regularly reviewed by the behavioral coach, who will document and report any AEs in accordance with UIC IRB guidelines.

### Control condition—stretching and flexibility program

This program mirrors the structure of the GEMS intervention program for aerobic and resistance exercise, but it focuses specifically on stretching and flexibility as the primary training modality (Fig. 1). The inclusion of this control condition is important for RCTs that provide Class I evidence supporting further intervention efforts. This program builds on a completed RCT aimed at improving mobility in MS and has been refined through ongoing RCTs targeting cognitive dysfunction in MS [46]. Participants in this control group will follow a 4-month, home-based stretching program that emphasizes flexibility as an essential fitness component, guided by *Stretching for People with MS: An Illustrated Manual* from the NMSS. They will receive all necessary equipment, including a yoga mat, a program manual, a logbook, a calendar, and a prescribed stretching regimen, designed to align with the resources provided in the intervention condition. To support adherence and motivation, participants will also receive periodic electronic newsletters that cover behavior-change principles, such as Outcome Expectations, Self-Monitoring, Goal-Setting, Self-Efficacy, and overcoming Barriers and Facilitators. Additionally, they will have regular video or telephone check-ins with an exercise specialist, offering motivation and social accountability. These newsletters and interactions will follow the same timeline and frequency as those in the GEMS intervention, but with a specific focus on flexibility and stretching. Safety and compliance will be actively monitored as in the intervention condition, and participants will receive guidance on maintaining safety throughout the program.

### Statistical methods and analysis

Data will be analyzed in SPSS using intention-to-treat principles, with specific analyses aligned to each study aim. For Aim 1, feasibility will be evaluated through process, resource, management, and scientific metrics using percentage, frequency analysis, and descriptive statistics. Aim 2 will focus on examining changes over time in physical function, cognitive function, fatigue, depression, and quality of life across conditions, analyzed through a two-way linear mixed model analysis of variance (condition by time). Effect sizes will be calculated with Cohen’s *d*, with *d* values of 0.2, 0.5, and 0.8 indicating small, medium, and large effects, respectively [47]. For Aim 3, the association among individual and structural SDOH and feasibility metrics will be analyzed using covariate analysis or regression models, with subgroup analyses further investigating the effects of SDOH factors on intervention outcomes.

### Publication plan

Our a-priori publication plan includes this protocol paper along with two additional papers. One will focus on analyzing the feasibility and efficacy of the exercise program, whereas the other will examine physical and cognitive outcomes in relation to SDOH variables.

### Trial status

The trial was registered on ClinicalTrials.gov in November 2023 (NCT05998616) and received UIC IRB approval that same month (IRB# 2023–0665). Participant recruitment began on March 18, 2024, and the first wave of participants started on April 22, 2024. Additional waves will follow at intervals of 1 to 2 months, with recruitment concluding on February 1, 2025.

## Discussion

We propose a study to evaluate the feasibility and preliminary efficacy of a remotely delivered exercise intervention for Hispanics/Latinos with MS, an underrepresented population in MS research. This pilot will use a targeted recruitment strategy to assess both the feasibility of conducting an exercise trial within this group and the intervention’s impact on health-related outcomes. By considering both individual and structural SDOH, this study aims to enhance the intervention’s real-world applicability and sustainability, ultimately identifying best practices for engaging this population. Additionally, this approach seeks to reveal unique barriers to physical activity participation and to achieve clinically meaningful improvements in health outcomes. Findings from this study may inform future exercise guidelines for the long-term management of MS symptoms, thereby enhancing the relevance and generalizability of MS research outcomes.

The study has several limitations that should be acknowledged. The small sample size is drawn from the Chicagoland community and nearby neighborhoods and may limit the generalizability of the findings to the broader Hispanic/Latino population with MS. Remote delivery of the intervention might affect participant adherence and engagement due to varied access to technology and internet connectivity, potentially introducing sampling bias or inaccuracies in self-reported exercise adherence data. Participants may not capture all AEs or compliance issues accurately, affecting the behavioral coach’s ability to monitor these aspects. A significant limitation is that this pilot is an English-only intervention and does not include Spanish-speaking-only participants. As a pilot study, we did not have sufficient funds to hire bilingual staff and translate all materials into Spanish. Moreover, this study does not fully address all individual differences within the Hispanic/Latino community, such as diverse regional, cultural, and geographical backgrounds. Finally, the short duration of the pilot study limits the ability to observe long-term effects or the sustainability of intervention outcomes. This pilot study is a necessary preliminary step to assess feasibility and initial efficacy before conducting a more comprehensive, long-term study, which could further evaluate the intervention’s sustained impact and the feasibility of expanding it to a bilingual format.

If this pilot study proves successful, the next steps will involve program modifications with fully translated materials (i.e., Spanish-only, Bilingual, and with regional/geographical cultural tailoring per each of the materials), bilingual behavioral coaches, and scaling up the intervention to a larger, more diverse sample to validate findings. This would also include conducting a fully powered Phase II RCT to assess the long-term efficacy and sustainability of the exercise program. We would explore integrating the intervention into clinical practice, potentially collaborating with healthcare providers to offer the program as part of standard care or follow up care for Hispanics/Latinos with MS. Further research could also investigate the impact of the intervention on other health outcomes and explore adaptations for different cultural heritages within the Hispanic/Latino community (i.e., Mexican, Puerto Rican, Cuban or another Hispanic, Latino, or Spanish origin). Overall, this study aims to address significant health disparities by providing a remotely delivered exercise intervention for Hispanics/Latinos with MS. The findings from this pilot study will offer valuable insights into potential strategies for managing MS in underrepresented populations using a comprehensive approach in healthcare and provide an inclusive model for future rehabilitation trials for other chronic health conditions.

## Supplementary Information

Supplementary Material 1: Appendix 1 : World Health Organization Trial Registration Data Set. Appendix 2: SPIRIT 2013 Checklist: Recommended items to address in a clinical trial protocol and related documents*.

## Data Availability

The datasets generated and analyzed during the current study are not publicly available due to the ongoing trial but will be available from the corresponding author on reasonable request.

## Abbreviations

30STS: 30 Second sit-to-stand
AE: Adverse event
CVLT-II: California Verbal Learning Test II
C3EN: Chicago Chronic Condition Equity Network
ENRL: Exercise Neuroscience Research Lab
FERLA MS: Feasibility and Efficacy of Exercise Remotely delivered for Hispanics/Latinos with MS
FSS: Fatigue Severity Scale
GLTEQ: Godin Leisure-Time Exercise Questionnaire
GEMS: Guidelines for Exercise in Multiple Sclerosis
HIPAA: Health Insurance Portability and Accountability Act
HADS: Hospital Anxiety and Depression Scale
IRB: Institutional Review Board
MS: Multiple sclerosis
NMSS: National Multiple Sclerosis Society
E: Number of exercises
RCT: Randomized controlled trial
RPE: Rate of perceived exertion
R: Repetitions
SAE: Serious adverse event
S: Sets
SF-12: Short-Form 12-Item Health Survey
SDOH: Social determinants of health
SCT: Social-cognitive theory
SDMT: Symbol Digit Modalities Test
US: United States
UIC: University of Illinois Chicago
