# Supplementary material for: Rationale for a 4-month, parallel-group, randomized controlled trial to assess the Feasibility and Efficacy of a Remotely delivered exercise training intervention for Hispanics/Latinos with Multiple Sclerosis (FERLA MS)
Source: Pilot Feasibility Stud. 2025 May 8;11:62. doi: 10.1186/s40814-025-01641-5 (PMC12060554; doi:10.1186/s40814-025-01641-5)
Supplement: Supplementary file 1 — Supplementary Material 1: Appendix 1 : World Health Organization Trial Registration Data Set. Appendix 2: SPIRIT 2013 Checklist: Recommended items to address in a clinical trial protocol and related documents*. [file 40814_2025_1641_MOESM1_ESM.zip › AppendixA_WHOChecklist.12.19.24.docx]

Appendix A: World Health Organization Trial Registration Data Set

| **Data** **category** | **Information** |
| --- | --- |
| Primary registry and trial identifying number | ClinicalTrials.gov (NCT05998616) |
| Date of registration in primary registry | 21 August 2023 |
| Source(s) of Monetary or Material Support | Chicago Chronic Condition Equity Network; National Institute of Minority Health and Disparities |
| Primary sponsor | University of Chicago; University of Illinois at Chicago (UIC), Chicago, Illinois |
| Contact for public queries | VAF; [vflores1@uic.edu](mailto:vflores1@uic.edu) |
| Contact for scientific queries | VAF; [vflores1@uic.edu](mailto:vflores1@uic.edu) |
| Public title | Feasibility of Exercise Remotely-delivered for Hispanics/Latinos with MS (FERLA MS) |
| Scientific title | The Feasibility and Efficacy of a Remotely Delivered Exercise Training Intervention for the Hispanic/Latino Community with Multiple Sclerosis |
| Countries of recruitment | United States of America |
| Health condition(s) or problem(s) studied | Multiple sclerosis (MS) |
| Intervention(s) | Exercise intervention: Home-based exercise training program (aerobic and resistance training) with embedded behavioral approaches guided by the behavioral coach, and using the Guidelines for Exercise in Multiple Sclerosis.  Attention/Social contact, active control: Home-based stretching and flexibility program with same embedded behavioral approaches guided by the behavioral coach, and using the National Multiple Sclerosis Society *Stretching for People with MS: An Illustrated Manual* |
| Key inclusion and exclusion criteria | (1) age between 18-65 years, (2) self-reported diagnosis of MS, (3) relapse-free for at least 30 days, (4) able to walk with or without an assistive device, (5) insufficient physical activity (i.e., not meeting current physical activity guidelines of 150 minutes of moderate to vigorous physical activity per week), (6) willingness to complete all required testing procedures, outcome questionnaires, and randomization, (7) self-identify as Hispanic/Latino, (8) able to speak, read, and understand English, (9) currently reside in Chicago, (10) access to the internet and email, and (11) safe for exercise based on Physical Activity Readiness Questionnaire |
| Study type | Interventional; parallel-group randomized controlled trial; random number sequence with concealed allocation; treatment-blinded outcomes assessor; Phase Ib |
| Date of first enrollment | Actual date of April 2024 |
| Sample size | Enrollment target of 50 participants; 23 participants have been enrolled |
| Recruitment status | Recruiting |
| Primary outcome(s) | Feasibility, determined by measures of process (recruitment and enrollment of 50 participants, retention rate of at least 80% of enrolled participants through the duration of the study, and adherence to the intervention of at least 75% per participant), resource (total time and cost requirements), management (researcher capacity in delivering the remote intervention and control), and scientific (detailed safety procedures and adverse events) |
| Key secondary outcomes | Potential effects of the intervention on health-related outcomes (physical and cognitive function, symptoms of fatigue, anxiety and depression, exercise behavior, and quality of life); potential association of social determinants of health (SDOH; economic stability, education, healthcare access, neighborhood and built environment, and social and community context) on the feasibility and efficacy of the remote intervention for improving health outcomes |
| Ethics review | Approved by UIC Institutional Review Board on September 13, 2023 |
| Completion date | Last data analyzed on June 30, 2025 |
| Summary results | Publication plan includes this protocol paper along with two additional papers. One will focus on analyzing the feasibility and efficacy of the exercise program, whereas the other will examine physical and cognitive outcomes in relation to SDOH variables, with planned dates after completion of analysis |
| IPD sharing statement | No, unless by reasonable request |
